# The plasma miR-125a, miR-361 and miR-133a are promising novel biomarkers for Late-Onset Hypogonadism

**DOI:** 10.1038/srep23531

**Published:** 2016-03-22

**Authors:** Yao-ping Chen, Ju Wang, Kai Zhao, Xue-jun Shang, Hui-qin Wu, Xing-rong Qing, Fang Fang, Yan Zhang, Jin Shang, Hong-gang Li, Hui-ping Zhang, Huang-tao Guan, Yuan-zhong Zhou, Yi-qun Gu, Wei-xiong Wu, Cheng-liang Xiong

**Affiliations:** 1 Family Planning Research Institute/Center of Reproductive Medicine, Tongji Medical College, Huazhong University of Science and Technology, Hangkong Road 13, Wuhan 430030, China; 2 Center of Reproductive Medicine, General Hospital of Ningxia Medical University, Shengli South Street 804, Yinchuan, Ningxia 750004, China; 3 Department of Histology and Embryology, School of Medicine, Shihezi University, North 2nd Road 59, Shihezi, Xinjiang 832002, China; 4 Department of Andrology, Jinling Hospital, School of Medicine, Nanjing University, East Zhongshan Road 305, Nanjing 210002, China; 5 Emergency Department, General Hospital of Ningxia Medical University, Shengli South Street 804, Yinchuan, Ningxia 750004, China; 6 Wuhan Tongji Reproductive Medicine Hospital, Sanyang Road 128, Wuhan 430013, China; 7 School of Public Health, Zunyi Medical University, Dalian Road 201, Zunyi, Guizhou 563099, China; 8 Key Laboratory of Male Reproductive Health, National Health and Family Planning Commission, National Research Institute for Family Planning, Da Hui Si Rd 12, Hai Dian District, Beijing 100081, China; 9 Guangzhou Institute for Population and Family Planning, Xin Shi Xin Da road 93, Baiyun District, Guangzhou 510410, China

## Abstract

Circulating miRNAs have been shown to serve as diagnostic/prognostic biomarkers in cancers and other diseases. However, the role of plasma miRNAs in Late-onset hypogonadism (LOH) diagnosis is still unknown. Using Illumina HiSeq2000 sequencing at discovery phase, and then two-step validated by reverse transcriptase polymerase chain reaction (RT-PCR) assays in verification phases. We verified that the expression levels of miR-125a-5p, miR-361-5p and miR-133a-3p were significantly altered in LOH group compared to the control group. The area under the receiver operating characteristic (ROC) curve (AUC) is 0.682, 0.698 and 0.765, respectively. The combination of three miRNAs showed a larger AUC (0.835) that was more efficient for the diagnosis of LOH. Among three miRNAs, miR-133a-3p had the best diagnostic value for LOH with 68.2% sensitivity and 77.3% specificity. Regression analyses show that miR-133a-3p level was negatively associated with the ageing males’ symptoms (AMS) scale. However, miR-361-5p level was positively associated with serum testosterone concentrations. In summary, plasma miRNAs are differentially expressed between LOH and healthy controls. We validated three miRNAs that could act as novel biomarkers for diagnosis of LOH. These miRNAs may be involved in the development of LOH. However, further large and functional studies are warranted to confirm our findings.

Late-onset hypogonadism (LOH) is characterised by a deficiency of the serum testosterone and a series of particular clinical symptoms that are associated with advancing age. It may result in significant alterations in the quality of life and adversely affect the function of multiple organ systems^1^. Recently, studies have demonstrated that LOH is associated with prostate cancer^2^, type 2 diabetes mellitus^3^, metabolic syndrome, obesity^4^, cardiovascular disease and mortality^5^. Moreover, population aging is a global phenomenon that will continue to affect all regions around the world. By 2050, there will be the same number of old (aged 60 or over) and young (under age 15) people in the world, with 2 billion people aged 60 or over^6^. With increasing life expectancy, the field of LOH is attracting increasing interest in the medical community and the public at large^7^.

For the diagnosis of LOH, the European Male Ageing Study (EMAS) has recently defined the strict diagnostic criteria for LOH to include the simultaneous presence of reproducibly low serum testosterone (total T < 11 nmol l^−1^ and free T < 220 pmol l^−1^) and three sexual symptoms (erectile dysfunction, and reduced frequency of sexual thoughts and morning erections). By these criteria, only 2.1% of 40–79 years old men have LOH. However, the prevalence of low testosterone is higher, which is 23.3% in 40–79 years men^8^. Moreover, the sexual symptoms have a high prevalence of 28–40% and not all men with sexual dysfunction report the symptoms to their doctor/investigator^9^. The testosterone deficiency and clinical symptoms do not coincide in the same individual. In addition, low testosterone is the main diagnosis biomarker for LOH. However, there are still problems that the secretion of testosterone is cyclical changing, and not all people are sensitive to receptors of testosterone^8^,^9^. The current diagnostic criteria do not completely satisfy the clinical requirements. So the definite and objective biomarkers are needed for the diagnosis of LOH.

Recently, microRNAs (miRNAs) have been showed as novel diagnostic biomarkers for many diseases due to their characteristics of stability in serum, economical, rapid and noninvasive. Notably, circulating miRNAs have been reported as promising biomarkers with great accuracy for aging^10^,^11^, cancer^12^,^13^, muscle diseases^14^,^15^ and neurodegenerative disorders^16^. Moreover, several studies have demonstrated that miRNAs were regulated by androgen, and miRNAs can regulate the adipogenic differentiation^17^, the differentiation and regeneration of old skeletal muscle with age^18^.

With advancing age, LOH is characterized with decreasing of lean mass, muscle size, and strength^8^,^9^, and treatment with androgen significantly increases muscle mass and strength^4^. In addition, obesity and overweight are more common causes for low testosterone than chronological age, and most of LOH patients were obese or overweight^8^,^19^. LOH is a clinical and biochemical phenotype that was associated with the change of adipocyte and muscle cell in the condition of low testosterone levels. So it is speculated that the expression of miRNA in patients with LOH may be different from normal physiological state.

In present study, we first intended to find the difference of plasma miRNA expression between LOH and healthy control, and identify plasma-based miRNA biomarkers for the diagnosis of LOH.

## Results

### Characteristics of study population

From 36 pairs of participants, cases with unreliable Cq value were excluded. 32 pairs of participants were included in the analysis (10 patients with LOH and 10 healthy controls in discovery and training phases, 22 LOH and 22 healthy controls in validation phase). No significant differences of age, height, weight, waist circumference, body mass index (BMI), smoking status and alcohol intake were found in discovery and training set (*P* = 0.573, 0.119, 0.778, 0.311, 0.271, 0.712, 0.648, respectively), or in validation set (*P* = 0.628, 0.233, 0.629, 0.613, 0.434, 0.525, 0.936, respectively). The characteristics of these men are shown in Table 1.

### Distinct circulating miRNA profilings of LOH vs normal control in discovery set

**Figure 1:**
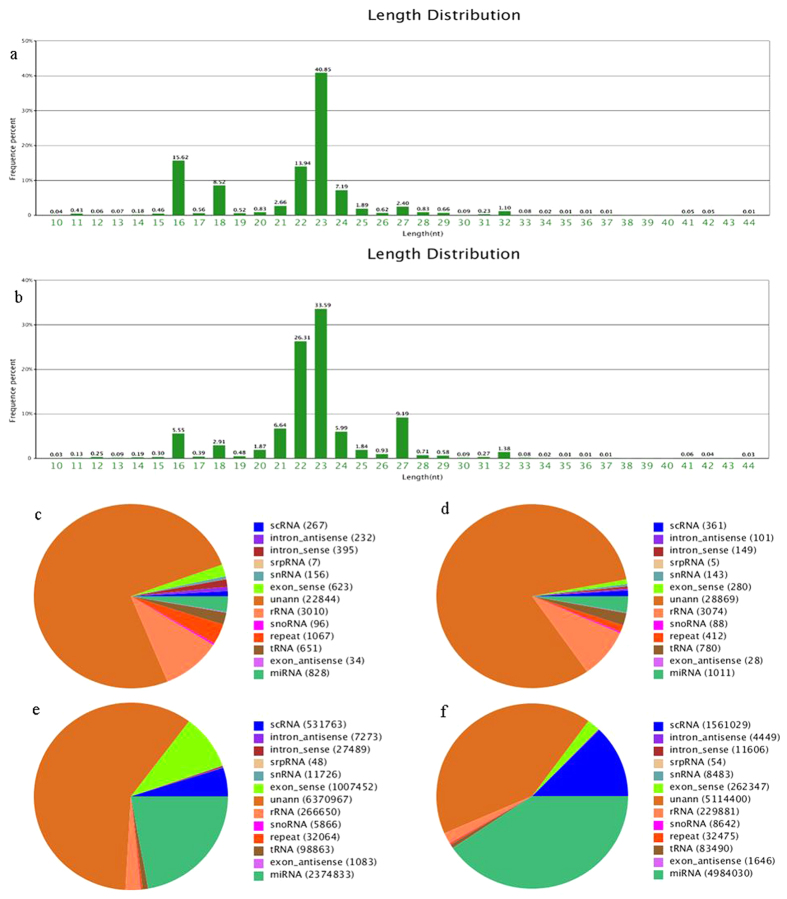
The circulating miRNAs signatures identified by Illumina Hiseq2000 sequencing. The length distribution and frequency percentages of the sequences identified in LOH (**a**) and control samples (**b**) RNA species (unique tags aligning) in LOH (**c**) and control samples (**d**) and RNA read counts (total tags aligning) in LOH samples (**e**) and control samples (**f**).

In total, genome-wide sequencing identified 13105774 and 13329645 raw reads in both LOH group and control group. As is shown in Fig. 1a,b, the dominant small RNAs were 22–23nt in length, accounting for 54.79% and 59.90% of the total reads in LOH and control group, respectively. After getting rid of low-quality sequences, sequences shorter than 18 nucleotides, and single-read sequences, 10736077 (82.18%) clean reads in LOH group and 12302532 (92.60%) clean reads in control group were remained for further analysis. Among these clean reads, 4384565 (57.03%) reads in LOH group and 7152503 (67.91%) reads in control group were perfectly mapped to the human genome in Genbank. Although miRNAs accounted only a tiny fraction of the total small RNAs, the expression levels of individual miRNAs were relatively high. Moreover, both the number of the unique miRNA sequences and the amount of miRNA species were mildly lower in LOH group compared with control group (828 vs 1011, 2374833 vs 4984030, respectively) (Fig. 1c–f).

The deep sequencing data and analyses of differentially expressed miRNAs were listed in Supplementary Table S1. Genome-wide sequencing showed that 239 miRNAs were differentially expressed between LOH group and control group. The miRNA levels were considered to be significantly different only if they met the following criteria: (1) having at least 10 copies in LOH or control groups; (2) showing a fold-change (log_2_LOH/control) >2 or <−2 between each comparisons (P < 0.05). According to these criteria, we found that 11 miRNAs were down-regulated (miR-125a-5p, miR-361-5p, miR-7849-3p, miR-150-5p, miR-877-5p, miR-133a-3p, miR-106b-5p, miR-335-5p, miR-381-3p, let-7e-5p and miR-505-3p) and 5 were up-regulated (miR-3615, miR-99a-5p, miR-148b-3p, miR-4433b-3p and miR-1301-3p) in LOH group compared to control group (Supplementary Table S1).

### Investigation of 16 selected single miRNAs using fluorescence quantitative RT-PCR (fq-RT-PCR) in training set

**Figure 2:**
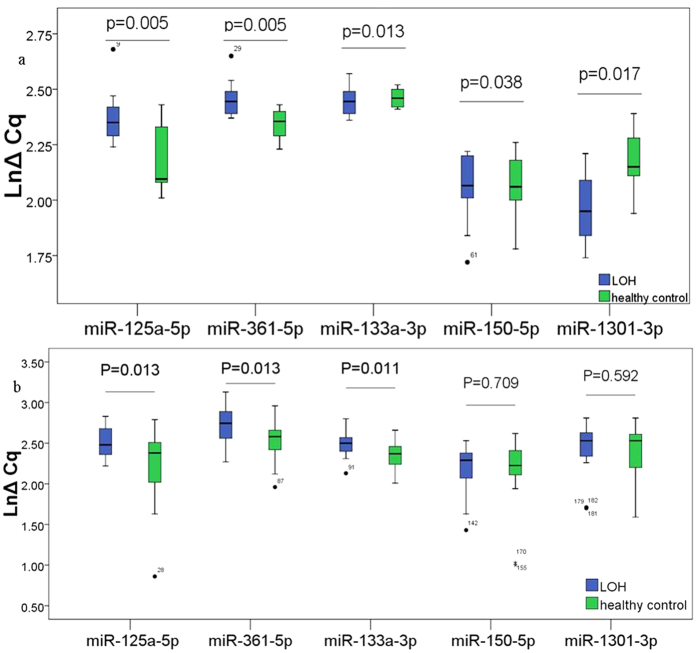
The different expressions of miRNAs are in LOH patients and healthy controls. The blue Boxplot represent LOH group, green Boxplot represent healthy control group. Expression levels of the miRNAs (LnΔCq scale at Y-axis) were normalized by the mean Cq value of let-7b-5p, let-7i-3p and U6 snRNA. Low expression level of the miRNA has a high LnΔCq scale. The line represents the median value. Wilcoxon signed-rank test was used to determine statistical significance. (**a**) Investigation of 16 selected single miRNAs using fq-RT-PCR in training set. The results showed that miR-125a-5p, miR-361-5p, miR-150-5p and miR-133a-3p were significantly decreased in LOH patients when compared with control group; while miR-1301-3p was increased in LOH patients. (**b**) Large-scale validation of the 5 miRNAs that were selected from the training set. The results showed that miR-125a-5p, miR-361-5p and miR-133a-3p were significantly decreased in LOH patients compared with healthy controls.

The expression levels of the 16 miRNAs selected by high-throughput sequencing were determined using fq-RT-PCR in a cohort of 10 LOH patients and 10 health control samples (Supplementary Table S2). The primers for real-time PCR of each miRNA were listed in Supplementary Table S3. MiRNA levels were normalized by the mean Cq value of let-7b-5p, let-7i-3p and U6 snRNA^13^,^18^,^20^,^21^. Only miRNAs with a Cq value <36, a detection rate >75% in both groups, and a p value < 0.05 were selected for further analyses. As a result, miR-125a-5p, miR-361-5p, miR-150-5p and miR-133a-3p were significantly decreased in LOH patients when compared with control group; while miR-1301-3p was increased in LOH patients (Fig. 2a). MiR-7849-3p, miR-381-3p and miR-505-3p displayed poor results of melting curving analysis; no significant difference was observed in the levels of miR-877-5p, miR-106-5p, miR-335-5p, let-7e-5p, miR-3615, miR-99a-5p, miR-148b-3p and miR-4433b-3p between LOH patients and control group (P > 0.05).

### Confirmation of 5 identified miRNAs using fq-RT-PCR in large-scale validation set

To further evaluate the diagnostic value of the 5 miRNAs (miR-125a-5p, miR-361-5p, miR-150-5p, miR-133a-3p and miR-1301-3p) that have been identified in the training phase, the expression levels of 5 miRNAs were measured on a total of 22 pairs plasma samples including 22 LOH patients and 22 healthy controls. The results showed that miR-125a-5p, miR-361-5p and miR-133a-3p were significantly down-regulated in LOH patients compared with healthy controls. However, there is no significant difference for miR-150-5p and miR-1301-3p (Fig. 2b).

**Figure 3:**
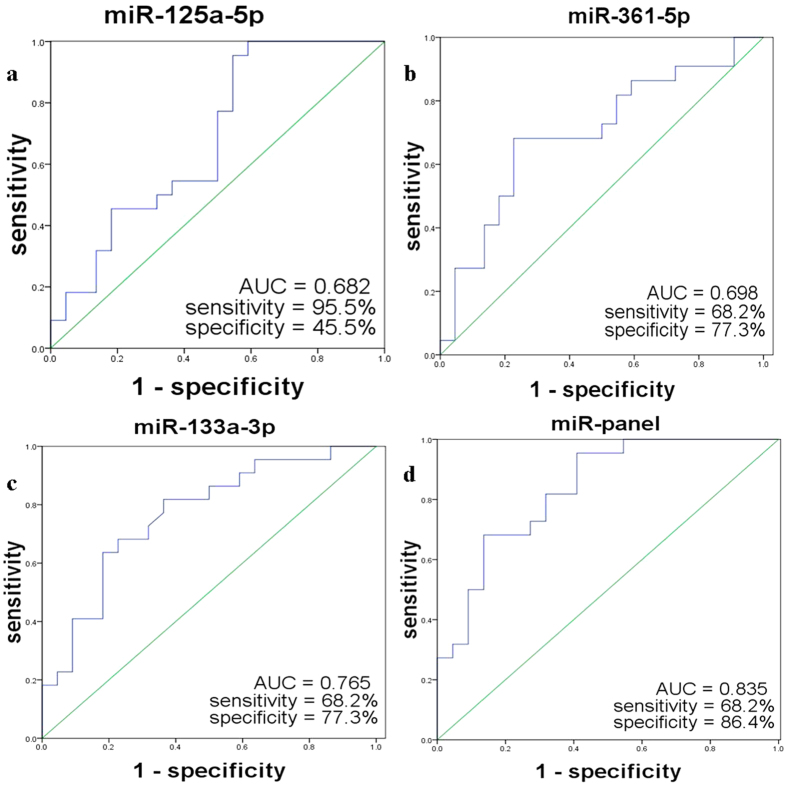
Receiver operating characteristic (ROC) curve analysis discriminated LOH from healthy control using 3 miRNAs and the miRNA panel that were selected in large-scale validation. AUC is area under the ROC curve.

ROC curve analyses indicated that miR-361-5p and miR-133a-3p were the more valuable biomarker for LOH compared with healthy controls with an AUC of 0.698 (95%CI: 0.540–0.857) and 0.765(95%CI: 0.624–0.907). The cut-off values of miR-361-5p and miR-133a-3p were 4.3800 and 1.7050, and the same optimal sensitivity and specificity were 68.2% and 77.3%, respectively. MiR-125a-5p was the less valuable biomarker for LOH compared with healthy controls with an AUC of 0.682 (95%CI: 0.521–0.842). The cut-off value of miR-125a-5p was −0.4900, the optimal sensitivity and specificity was 95.5% and 45.5%, respectively (Fig. 3a–c).

Furthermore, we combined these miRNAs to form different panels, and evaluated the diagnostic value of the panels. The miR-125a-5p/miR-361-5p/miR-133a-3p combination (miR-panel) showed better diagnostic value than other combinations with a AUC of 0.835 (95%CI: 0.717–0.952; sensitivity: 68.2%, specificity: 86.4%) (Fig. 3d), indicating an additive effect of the 3 miRNAs.

In addition, multivariate logistic regression analyses on variables including age, smoking status, alcohol intake and BMI revealed that miR-125a-5p, miR-361-5p, miR-133a-3p was a potential biomarker for the diagnosis of LOH (*P* = 0.029, 0.030, 0.005), with odds ratios (ORs) of 1.317 (95%CI: 1.029–1.686), 1.292 (95%CI: 1.025–1.630) and 1.902 (95%CI: 1.210–2.987), respectively.

### The relationships between plasma level of miR-125a-5p, miR-361-5p and miR-133a-3p with clinical characteristics

**Figure 4:**
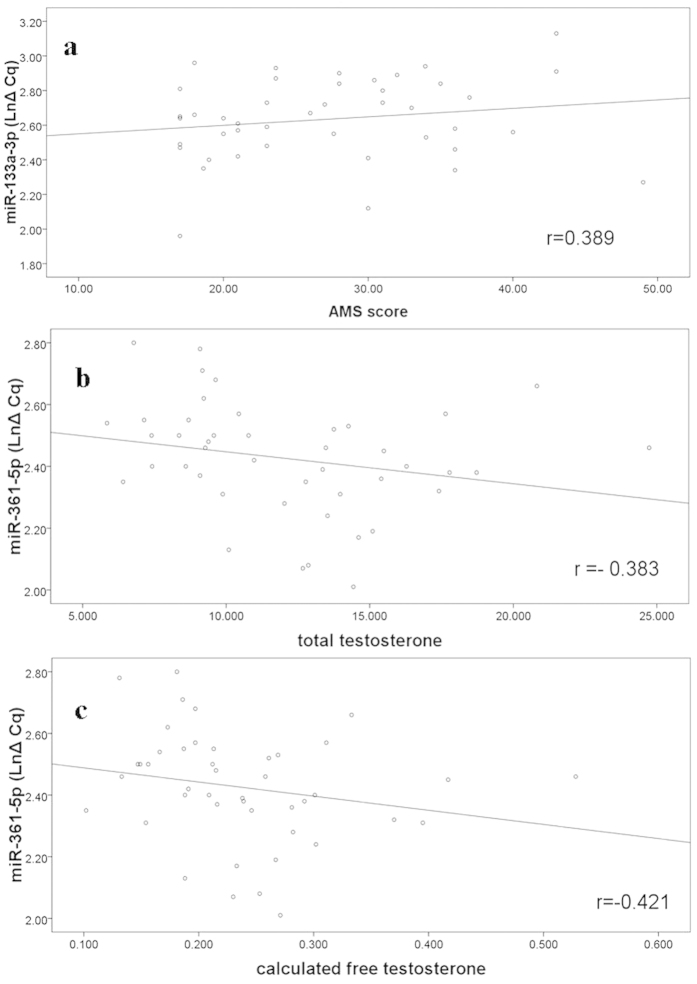
The relationships between plasma level of miR-125a-5p, miR-361-5p and miR-133a-3p with clinical characteristics. Expression levels of miRNAs (LnΔCq scale at Y-axis) were normalized by the mean Cq value of let-7b-5p, let-7i-3p and U6 snRNA. The results show that miR-133a-3p level was significantly associated with AMS (r = −0.389, p = 0.010) (**a**), and there were significant associations between miR-361-5p with the serum total testosterone and calculated free testosterone concentrations (r = 0.383 and r = 0.421, p = 0.010 and p = 0.004) (**b,c**).

Regression analysis was performed to investigate the association between the expression level of miR-125a-5p, miR-361-5p, miR-133a-3p with clinical and biochemical parameters. We found that miR-133a-3p level was significantly associated with the ageing males’ symptoms scale (AMS) (r = −0.389, p = 0.010), indicating that the expression level of miR-133a-3p was negatively associated with clinical symptoms severity. Interestingly, we observed that miR-361-5p was significantly associated with serum total testosterone and calculated free testosterone concentrations (r = 0.383 and r = 0.421, p = 0.010 and p = 0.004) (Fig. 4a–c).

## Discussion

In the present study, for the first time, plasma miRNA expression profiles of LOH patients were analyzed and the specifically modulated miRNAs in LOH patients were identified. Furthermore, ROC analysis indicated that miR-125a-5p, miR-361-5p and miR-133a-3p may serve as novel biomarkers for diagnosis of LOH. In addition, we found that there was positive association between the plasma level of miR-361-5p and serum testosterone concentration, and there was negative association between the plasma level of miR-133a-3p with AMS score. The expression level of miR-133a-3p was negatively associated with clinical symptoms severity.

Low testosterone level is the main diagnosis biomarker for LOH, but it cannot fully meet the clinical needs. Recently, miRNAs have gained significant attention. Studies had reported that miRNAs are stable in serum/plasma, and the test of miRNAs is convenient, rapid, economical and noninvasive. Furthermore, the development of high-throughput sequencing has given a significant boost to the search for miRNAs as biomarkers. Serum/plasma miRNAs have been proposed as novel biomarkers for the diagnosis of several diseases^12^,^13^,^16^. In recent years, there are researches on the testosterone^17^, testis^22^, aging^10^,^11^ and reproductive endocrine^23^. Which provide evidence that LOH is associated with the change of miRNAs. However, the plasma miRNAs profile in LOH has not been reported.

Our study identified 11 miRNAs down-regulated and 5 miRNAs up-regulated in the LOH patients compared to healthy controls in the discovery stage, and validated three miRNAs (miR-125a-5p, miR-361-5p and miR-133a-3p) by RT-qPCR eventually.

Among them, miR-125a has been reported to be a tumor suppressor in malignancies of the breast, ovary, lung, gastrointestinal, cervical and central nervous system^24^,^25^,^26^. Recent studies show that miR-125a linked to cardiomyopathy, and were identified as potential biomarkers for endothelial dysfunction^27^. MiR-361-5p was also reported to suppress proliferation, migration and invasion of cancer cells in colorectal carcinoma and gastric cancer. MiR-361-5p functions as a tumor-suppressive miRNA through directly binding to staphylococcal nuclease domain containing-1 (SND1)^28^. Furthermore, miR-361 was significantly associated with disease-free survival and distant metastasis in oropharyngeal carcinoma^29^. Moreover, the study reported that miR-361-regulated prohibitin inhibits mitochondrial fission and apoptosis and protects heart from ischemia injury^30^. As for miR-133a-3p, the studies demonstrated that miR-133a suppresses the migration and invasion of esophageal cancer cells by targeting the EMT regulator SOX4^31^. Down-regulation of miR-133a is associated with unfavorable prognosis in patients suffering from osteosarcoma^32^. And miR-133a is potently suppressed in metastatic prostate cancer^33^. Furthermore, miR-133a down-regulation is associated with cardiac fibrosis^14^. And miR-133a clearly improved cardiac function in myocardial infarction model by reducing fibrosis and hypertrophy and increasing vascularization and cardiomyocyte proliferation^34^.

Our study demonstrated that the plasma miR-125a, miR-361 and miR-133a down-regulated in the LOH compared to controls. The result is consistent with the results that LOH is associated with cancer, obesity and cardiovascular disease^2^,^4^,^5^. The result is consistent with the results that the plasma miR-125a, miR-361 and miR-133a down-regulated patients are susceptible to LOH, and LOH patients had a higher risk of CVD mortality and a higher risk of death from cancer compared with men without LOH^5^. The plasma miR-125a, miR-361 and miR-133a down-regulation is a signal in bad health. In our study we observed that miR-133a-3p level was significantly associated with AMS (r = −0.388) (Fig. 4a), indicating that the expression level of miR-133a-3p was negatively associated with clinical symptoms severity.

In addition, miR-133a is known as a muscle-specific microRNA, which promotes muscle maturation. Todaka *et al*.^35^ demonstrated that miR-133a was significantly decreased in the skeletal muscle resulting in development of skeletal muscle atrophy and centronuclear muscle fibers. Those correspond with the results that LOH is characterized by decrease in lean mass, muscle size, and strength with advancing age^8^,^9^, and treatment with androgen produce significant increases in muscle mass and strength^4^.

Interestingly, we observed that miR-361-5p was significantly associated with serum total testosterone and calculated free testosterone concentrations (r = 0.385 and r = 0.421, p = 0.010 and p = 0.004). Further research on the exact molecular mechanisms will be needed.

Finally, bioinformatics analyses predicted the potential targets by miR-125a, miR-361 and miR-133a using the miRNA target prediction databases (miRanda). A very complicated network of target genes are presented, and we will do further research on the specific pathways.

With several advantages, the result of our study is rational and reliable for future studies. Firstly, according to the EMAS rigorous inclusion/exclusion diagnostic criteria for LOH, LOH patients and 1:1 matched normal controls (matched based on age, BMI, smoking status, alcohol intake and living area) were selected from the random population of 6898 males. Secondly, we employed a widely utilized approach: a high-throughput sequencing screening followed by two-step fq-RT-PCR validation. Thirdly, we combined let-7b-5p, let-7i-3p and U6 snRNA as a normalization strategy to replace the traditional tissue approach, which may have a more accurate and reliable serum miRNAs expression. Moreover, in order to determine the accuracy of fq-RT-PCR in our experiment, we set up two negative controls per test and had detected the repeatability of the instrument by measuring the samples in triplicates. However, certain limitations still need to be addressed and should be mentioned. In our study, during screening phase, we pooled the serum of 10 patients and 10 controls, respectively, and during two-step validation phase, we had tested 10 pairs and 26 pairs, respectively. The sample size of the test is limited. So, large-scale prospective cohort studies in different ethnic populations are necessary to verify our findings. Furthermore, the exact molecular mechanisms and roles of these miRNAs remain unknown, and more fundamental studies will be needed.

In conclusion, we first performed an investigation of circulating miRNAs in LOH. In the study, we identified three serum miRNAs to distinguish LOH patients from healthy controls. Among these miRNAs, miR-361-5p and miR-133a-3p has better potential to discriminate LOH from healthy controls with the same sensitivity (68.2%) and specificity (77.3%), respectively. Our results contribute to the new avenue of miRNA pathology in LOH and may provide a useful tool for LOH diagnosis and treatment. Thus, future studies with larger sample size will be needed to confirm and extend our findings in other populations, and more epidemiological and functional studies are also needed to validate our findings.

## Materials and Methods

### Study population

This study was approved by the Ethical Committee Review Board of Tongji Medical College, Huazhong University of Science and Technology, China (S073). The methods were carried out in accordance with the approved guidelines. All of the participants who were enrolled in the study gave their written informed consent^7^.

We performed age-stratified, random sampling of men participating in the 12th Five-Year Plan of National Science and Technology of China (2012BAI32B03) in six areas of China: Hebei, Shanxi, Guangdong, Hubei, Jiangsu and Guizhou. Men between the ages of 18 and 89 years were invited to undergo a health assessment by questionnaire, physical examination, and blood tests for biochemical and hormone measurements. A total of 6898 men were recruited without the use of specific exclusion criteria^7^.

According to the EMAS diagnostic criteria for LOH^8^, inclusion criteria of LOH are who must have three moderate or above sexual symptoms (erectile dysfunction, and reduced frequency of sexual thoughts and morning erections), and total testosterone <11 nmol/L and calculated free testosterone <220 pmol/L, respectively. And co-morbidities were ruled out or current use of medications that could affect metabolism of sex hormones. 36 men (aged 40–65 years) with LOH and 1:1 normal controls (matched age, BMI, living area, smoking status and alcohol intake) were selected from 6898 males.

In the discovery phase, we subjected pooled plasma samples from 10 LOH patients and 10 healthy controls to Illumina HiSeq 2000 technology to select differential expression of miRNAs. Subsequently, we confirmed the differential expression of miRNAs by a two-step experimental procedure using fq-RT-PCR assays. In the training phase, we analyzed plasma samples from the 10 LOH patients and 10 healthy controls that had been assessed by Illumina HiSeq 2000 technology. In the validation phase, we analyzed additional plasma samples from 26 LOH patients and 26 healthy controls.

### Questionnaires

We collected data on general health status, medical conditions, medications, medical history, and lifestyle with the use of questionnaires. Interviewer-assisted questionnaires that were administered included the Medical Outcomes Study 36-Item Short-Form Health Survey (SF-36), the Beck Depression Inventory, and the simplified Chinese version of the aging males’ symptoms scale (CN-AMS). A detailed description of the study has been reported previously^7^,^36^.

### Sample collection and processing

Ten millilitres (10 ml) of fasting blood samples was collected between 08:00 h and 10:00 h on the day of screening, with five millilitres (5 ml) stored in an ethylene diamine tetra acetic acid (EDTA) vial and five millilitres (5 ml) stored in a vacuum drying vial^7^.

### Clinical and Sex steroids assessments

Height, weight, body-mass index (BMI), and waist circumference were measured. The total testosterone, sex hormone-binding globulin (SHBG) and luteinising hormone (LH) levels were measured using a Beckman DXI 800 Analysis System, and serum albumin was measured using a Roche Cobas C311 analyser, as described previously^7^,^36^. The level of calculated free testosterone (CFT) was calculated according to the Vermeulen *et al*. formula^37^.

### Plasma small RNA library construction and sequencing

We mixed 2 ml of each plasma samples from 10 LOH patients and 10 healthy controls separately. Total RNA of each mixed samples was isolated using TRIzol^®^ LS Reagent (Ambion) according to the protocol of manufacturer. And then checked the integrity and concentration of total RNA using Agilent 2100 Bioanalyzer (Agilent Technologies, USA) and checked the purity of total RNA at 260–280 nm absorbance using the NanoDrop Lite Spectrophotometer (Thermo, Germany). According to the TruSeq Small RNA Sample Pre Kit (Illumina), we prepare the libraries.

1. Filter Small RNA: Using the 200 ng-1 μg of RNA sample, then separate RNA segment of different size by PAGE gel, select 18–30nt (14–30 ssRNA Ladder Marker, TAKARA) stripe and recycle.
2. Adaptor ligation: Prepare connection 3′ adaptor system (TruSeq Small RNA Sample Pre Kit , Illumina)(Reaction condition: 70 °C for 2 min; 28 °C for 1 h; 28 °C for 15 min); Secondly adding 5′ adaptor mix system (Reaction condition: 70 °C for 2 min; 28 °C for 1 h).
3. RT-PCR: Prepare First Strand Master Mixand Super Script II (Invitrogen) reverse transcription (Reaction condition: 70 °C for 2 min; 50 °C for 1 h); Several rounds of PCR amplification with PCR Primer Cocktail and PCR Master Mix are performed to enrich the cDNA fragments (Reaction condition: 98 °C for 30s; 11 cycles of (98 °C for 10s, 60 °C for 30s, 72 °C for 15s); 72 °C for 10 min; 4 °C hold).
4. Purify PCR products: Then the PCR products are purified with PAGE gel, dissolve the recylced products in EB solution.
5. Validation of the Library: The final library was quantitated in two ways. Determine the average molecule length using the Agilent 2100 bioanalyzer instrument (Agilent DNA 1000 Reagents), and quantify the library by real-time quantitative PCR (QPCR) (TaqMan Probe).
6. Sequencing Libraries: Finally, the qualified libraries will amplify on cBot to generate the cluster on the flowcell. And the amplified flowcell will be sequenced single end on the HiSeq 2000 System (Illumina), read length 50 are the most frequently used sequencing strategy at BGI according to the protocol of manufacturer.

### The quantitative validation of miRNAs by fq-RT-PCR

The total RNA (20 ul solution) was extracted from 250 ul plasma of each sample using the TRIzol^®^ LS Reagent (Ambion) according to the protocol of manufacturer. Then the total RNA was reversely transcribed into cDNA (20 ul solution), and fq-RT-PCR was conducted for each sample using NCode™ EXPRESS SYBR^®^ GreenER™ miRNA qRT-PCR Kit Universal (Invitrogen Corporation, USA) and Light Cycler 96^®^ Real-Time PCR System (Roche, Germany) in a final 20 ul reaction volume according to the protocol of manufacturer. At the end of PCR cycles, melting curve analyses were performed to validate the specific generation of the expected PCR products. The Cq uniformity (SD < 0.2) of Real-Time PCR System had been detected by measuring the samples in triplicates. In order to avoid test errors that were caused by pollution we set up two negative controls per test. All miRNA primers were purchased from GENEWIZ (Suzhou, China).

### Statistical analysis and Quality control

All statistical analyses were performed using Statistical Product and Service Solutions 13.0 (SPSS Inc., Chicago, IL, USA). Continuous variables are expressed as the means ± SE, and an analysis of variance (ANOVA) or t-test was used to calculate the difference between groups along with Bonferroni’s method for multiple comparisons. Count data are expressed as the percentages, and an x^2^ test (Pearson Chi-square) was used to calculate the difference between the groups.

The expression levels of miRNAs for fq-RT-PCR were normalized by the mean Cq value of three miRNAs (let-7b, let-7i and U6 snRNA)^13^,^18^,^20^,^21^, and were calculated utilizing the 2^−ΔΔCt^ method. Expression differences of miRNAs were compared using the Wilcoxon signed-rank test. Receiver operator characteristic (ROC) curves, area under the ROC curve (AUC) and multivariate logistic regression analyses were established to evaluate the diagnostic value of plasma miRNAs for LOH group and control group, and odds ratios (ORs) [95% confidence intervals (CI)] were calculated in the additive model to assess the strength of the associations. Statistical significance was defined as a p-value < 0.05.

## Additional Information

**How to cite this article**: Chen, Y.-p. *et al*. The plasma miR-125a, miR-361 and miR-133a are promising novel biomarkers for Late-Onset Hypogonadism. *Sci. Rep*. **6**, 23531; doi: 10.1038/srep23531 (2016).

## Supplementary Material

Supplementary Information

## Figures and Tables

**Table 1:** Characteristics of the study population*.

Variable	Discovery and training set			Validation set		
	LOH	control	*P*	LOH	control	*P*
No.	10	10		22	22	
Age — yr	53.4 ± 1.8	52.1 ± 1.4	0.573	53.4 ± 1.4	54.3 ± 1.4	0.628
Height — cm	164.6 ± 1.2	167.9 ± 1.6	0.119	163.6 ± 2.6	167.0 ± 1.1	0.233
Weight — kg	72.5 ± 1.9	71.8 ± 1.6	0.778	72.1 ± 1.8	73.2 ± 1.3	0.629
Body-mass index†	26.8 ± 2.8	25.5 ± 2.3	0.271	27.6 ± 7.1	26.3 ± 2.3	0.434
Waist circumference — cm	94.5 ± 2.7	90.6 ± 2.6	0.311	93.4 ± 1.9	92.1 ± 1.5	0.613
Smoking status — no./total no. (%)						
Never smoked	5(50.0%)	5(50.0%)	0.712	7(31.8%)	8(36.4%)	0.525
Current smoker	5(50.0%)	4(40.0%)		15(68.2%)	12(54.5%)	
Former smoker	0(0%)	1(10.0%)		0(0%)	2(9.1%)	
Alcohol intake — no./total no. (%)						
None	4(40.0%)	3(30.0%)	0.648	5(22.7%)	6(27.3%)	0.936
1–4 days/wk	2(20.0%)	2(20.0%)		6(27.3%)	7(31.8%)	
≥5 days/wk	4(40.0%)	5(50.0%)		11(50.0%)	9(40.9%)	
Body-mass index — no./total no. (%)						
<24	1(10.0%)	3(30.0%)	0.328	4(18.2%)	4(18.2%)	0.766
≥24 to <28	6(60.0%)	5(50.0%)		11(50.0%)	13(59.1%)	
≥28	3(30.0%)	2(20.0%)		7(31.8%)	5(22.7%)	
AMS Score (low score favorable; range, 17–85)	31.1 ± 1.9	20.2 ± 1.4	<0.001	33.3 ± 1.4	20.9 ± 1.0	<0.001
Total Testosterone — nmol/L	8.9 ± 0.5	15.3 ± 0.9	<0.001	8.8 ± 0.3	15.5 ± 0.6	<0.001
Sex hormone-binding globulin — nmol/L	30.0 ± 4.4	41.9 ± 3.2	0.045	32.2 ± 2.7	37.9 ± 2.3	0.118
Free Testosterone — pmol/L	186.5 ± 8.6	272.1 ± 11.6	<0.001	176.9 ± 6.6	299.0 ± 15.4	<0.001
luteinizing hormone (IU/L)	7.0 ± 1.0	4.9 ± 0.5	0.096	6.4 ± 0.6	5.3 ± 0.4	0.150

^*^Measurement data are expressed as the means ± SE, and an analysis of variance (ANOVA) was used to calculate the difference between the groups. Count data are expressed as the percentages, and a x^2^ test (Pearson Chi-square) was used to calculate the difference between the groups.

^†^The body-mass index is the weight in kilograms divided by the square of the height in meters.
