# Supplementary material for: The plasma miR-125a, miR-361 and miR-133a are promising novel biomarkers for Late-Onset Hypogonadism
Source: Sci Rep. 2016 Mar 22;6:23531. doi: 10.1038/srep23531 (PMC4802305; doi:10.1038/srep23531)
Supplement: Supplementary Information [file srep23531-s1.doc]

**The plasma miR-125a, miR-361 and miR-133a are promising novel biomarkers for Late-Onset Hypogonadism.**

Yao-ping Chen^1★^, Ju Wang^1,2^, Kai Zhao^1^, Xue-jun Shang^3^, Hui-qin Wu^4^, Xing-rong Qing^1^, Fang Fang^1^, Yan Zhang^1^, Jin Shang^1^, Hong-gang Li^1,5^, Hui-ping Zhang^1,5^, Huang-tao Guan^1,5^, Yuan-zhong Zhou^6^, Yi-qun Gu^7^,Wei-xiong Wu^8^, Cheng-liang Xiong ^1,5★^_。_

^1^Family Planning Research Institute/Center of Reproductive Medicine, Tongji Medical College, Huazhong University of Science and Technology, Hangkong Road 13, Wuhan 430030, China;

^2^Department of Histology and Embryology, School of Medicine, Shihezi University, North 2nd Road 59, Shihezi, Xinjiang 832002, China;

^3^Department of Andrology, Jinling Hospital, School of Medicine, Nanjing University, East Zhongshan Road 305, Nanjing 210002, China;

^4^Emergency Department, General Hospital of Ningxia Medical University, Shengli South Street 804, Yinchuan, Ningxia 750004, China;

^5^Wuhan Tongji Reproductive Medicine Hospital, Sanyang Road 128, Wuhan 430013, China;

^6^School of Public Health, Zunyi Medical University, Dalian Road 201, Zunyi, Guizhou 563099, China;

^7^Key Laboratory of Male Reproductive Health, National Health and Family Planning Commission, National Research Institute for Family Planning, Da Hui Si Rd 12, Hai Dian District, Beijing 100081, China;

^8^Guangzhou Institute for Population and Family Planning, Xin Shi Xin Da road 93, Baiyun District, Guangzhou 510410, China.

**Table S1. Differentially expressed miRNAs in LOH samples compared to health control samples by Illumina Hiseq2000 sequencing*.**

| pairwise | miR-name | A-expressed | B-expressed | A-std | B-std | fold-change(log2 B/A) | p-value | sig-lable |
| --- | --- | --- | --- | --- | --- | --- | --- | --- |
| A-B | hsa-miR-3615 | 69 | 13 | 15.9187 | 1.8184 | -3.12998103 | 6.02E-18 | ** |
| A-B | hsa-miR-4433b-3p | 582 | 169 | 134.2706 | 23.6395 | -2.50587202 | 2.97E-109 | ** |
| A-B | hsa-miR-99a-5p | 199 | 62 | 45.9104 | 8.6725 | -2.40430116 | 1.06E-36 | ** |
| A-B | hsa-miR-148b-3p | 12878 | 4768 | 2971.0243 | 666.9423 | -2.15532655 | < 0.001 | ** |
| A-B | hsa-miR-1301-3p | 469 | 182 | 108.2008 | 25.4579 | -2.08752584 | 1.97E-70 | ** |
| A-B | hsa-miR-150-5p | 119 | 889 | 27.4539 | 124.3523 | 2.17935018 | 2.73E-76 | ** |
| A-B | hsa-miR-106b-5p | 65 | 507 | 14.9959 | 70.9186 | 2.24159595 | 1.16E-45 | ** |
| A-B | hsa-miR-125a-5p | 555 | 4514 | 128.0415 | 631.4131 | 2.30197271 | < 0.001 | ** |
| A-B | hsa-miR-335-5p | 57 | 525 | 13.1502 | 73.4364 | 2.4814106 | 6.42E-53 | ** |
| A-B | hsa-miR-877-5p | 85 | 788 | 19.61 | 110.2245 | 2.49078349 | 5.81E-79 | ** |
| A-B | hsa-miR-505-3p | 49 | 459 | 11.3046 | 64.2044 | 2.50576223 | 5.99E-47 | ** |
| A-B | hsa-miR-7849-3p | 127 | 1612 | 29.2996 | 225.4847 | 2.94407667 | 4.41E-190 | ** |
| A-B | hsa-miR-133a-3p | 73 | 1014 | 16.8415 | 141.8371 | 3.0741424 | 3.17E-125 | ** |
| A-B | hsa-miR-361-5p | 253 | 3716 | 58.3685 | 519.7898 | 3.15466643 | < 0.001 | ** |
| A-B | hsa-let-7e-5p | 52 | 765 | 11.9967 | 107.0073 | 3.1569997 | 3.32E-97 | ** |
| A-B | hsa-miR-381-3p | 53 | 822 | 12.2274 | 114.9804 | 3.23319838 | 1.41E-106 | ** |
| A-B | hsa-miR-2467-5p | 188 | 0 | 43.3726 | 0.01 | -12.08256826 | 2.13E-80 | ** |
| A-B | hsa-miR-33a-5p | 171 | 0 | 39.4506 | 0.01 | -11.94583135 | 3.33E-73 | ** |
| A-B | hsa-miR-487b-3p | 136 | 0 | 31.3759 | 0.01 | -11.61544093 | 2.15E-58 | ** |
| A-B | hsa-miR-203a-3p | 135 | 0 | 31.1452 | 0.01 | -11.60479396 | 5.68E-58 | ** |
| A-B | novel_mir_1 | 128 | 0 | 29.5303 | 0.01 | -11.52798044 | 5.21E-55 | ** |
| A-B | hsa-miR-330-3p | 126 | 0 | 29.0689 | 0.01 | -11.50526062 | 3.66E-54 | ** |
| A-B | hsa-miR-378a-5p | 126 | 0 | 29.0689 | 0.01 | -11.50526062 | 3.66E-54 | ** |
| A-B | hsa-miR-942-3p | 124 | 0 | 28.6075 | 0.01 | -11.48217764 | 2.57E-53 | ** |
| A-B | hsa-miR-324-3p | 121 | 0 | 27.9154 | 0.01 | -11.44684555 | 4.77E-52 | ** |
| A-B | novel_mir_2 | 95 | 0 | 21.917 | 0.01 | -11.0978347 | 4.79E-41 | ** |
| A-B | hsa-miR-487a-5p | 88 | 0 | 20.3021 | 0.01 | -10.9874132 | 4.39E-38 | ** |
| A-B | hsa-miR-580-3p | 78 | 0 | 17.995 | 0.01 | -10.81338044 | 7.47E-34 | ** |
| A-B | novel_mir_3 | 78 | 0 | 17.995 | 0.01 | -10.81338044 | 7.47E-34 | ** |
| A-B | hsa-miR-432-3p | 76 | 0 | 17.5336 | 0.01 | -10.7759066 | 5.24E-33 | ** |
| A-B | hsa-miR-485-5p | 76 | 0 | 17.5336 | 0.01 | -10.7759066 | 5.24E-33 | ** |
| A-B | hsa-miR-659-5p | 76 | 0 | 17.5336 | 0.01 | -10.7759066 | 5.24E-33 | ** |
| A-B | hsa-miR-2355-3p | 75 | 0 | 17.3029 | 0.01 | -10.75679815 | 1.39E-32 | ** |
| A-B | hsa-miR-3620-3p | 74 | 0 | 17.0722 | 0.01 | -10.73743324 | 3.68E-32 | ** |
| A-B | hsa-miR-376b-3p | 74 | 0 | 17.0722 | 0.01 | -10.73743324 | 3.68E-32 | ** |
| A-B | hsa-miR-1193 | 72 | 0 | 16.6108 | 0.01 | -10.69790576 | 2.58E-31 | ** |
| A-B | hsa-miR-195-5p | 72 | 0 | 16.6108 | 0.01 | -10.69790576 | 2.58E-31 | ** |
| A-B | hsa-miR-1180-3p | 71 | 0 | 16.3801 | 0.01 | -10.67772841 | 6.84E-31 | ** |
| A-B | hsa-miR-629-5p | 70 | 0 | 16.1494 | 0.01 | -10.65726474 | 1.81E-30 | ** |
| A-B | hsa-miR-3136-5p | 68 | 0 | 15.688 | 0.01 | -10.61544569 | 1.27E-29 | ** |
| A-B | novel_mir_4 | 68 | 0 | 15.688 | 0.01 | -10.61544569 | 1.27E-29 | ** |
| A-B | novel_mir_5 | 68 | 0 | 15.688 | 0.01 | -10.61544569 | 1.27E-29 | ** |
| A-B | hsa-miR-3677-3p | 67 | 0 | 15.4573 | 0.01 | -10.59407266 | 3.37E-29 | ** |
| A-B | hsa-miR-5001-3p | 67 | 0 | 15.4573 | 0.01 | -10.59407266 | 3.37E-29 | ** |
| A-B | hsa-miR-6753-3p | 67 | 0 | 15.4573 | 0.01 | -10.59407266 | 3.37E-29 | ** |
| A-B | novel_mir_6 | 67 | 0 | 15.4573 | 0.01 | -10.59407266 | 3.37E-29 | ** |
| A-B | novel_mir_7 | 67 | 0 | 15.4573 | 0.01 | -10.59407266 | 3.37E-29 | ** |
| A-B | hsa-miR-338-5p | 66 | 0 | 15.2266 | 0.01 | -10.57237819 | 8.93E-29 | ** |
| A-B | hsa-miR-409-5p | 66 | 0 | 15.2266 | 0.01 | -10.57237819 | 8.93E-29 | ** |
| A-B | hsa-miR-4732-5p | 66 | 0 | 15.2266 | 0.01 | -10.57237819 | 8.93E-29 | ** |
| A-B | novel_mir_8 | 65 | 0 | 14.9959 | 0.01 | -10.55035248 | 2.37E-28 | ** |
| A-B | hsa-let-7i-3p | 64 | 0 | 14.7651 | 0.01 | -10.52797532 | 6.27E-28 | ** |
| A-B | hsa-miR-379-3p | 64 | 0 | 14.7651 | 0.01 | -10.52797532 | 6.27E-28 | ** |
| A-B | hsa-miR-6735-3p | 64 | 0 | 14.7651 | 0.01 | -10.52797532 | 6.27E-28 | ** |
| A-B | novel_mir_9 | 64 | 0 | 14.7651 | 0.01 | -10.52797532 | 6.27E-28 | ** |
| A-B | hsa-miR-2277-5p | 63 | 0 | 14.5344 | 0.01 | -10.5052558 | 1.66E-27 | ** |
| A-B | hsa-miR-376a-3p | 63 | 0 | 14.5344 | 0.01 | -10.5052558 | 1.66E-27 | ** |
| A-B | hsa-miR-6859-5p | 63 | 0 | 14.5344 | 0.01 | -10.5052558 | 1.66E-27 | ** |
| A-B | novel_mir_10 | 63 | 0 | 14.5344 | 0.01 | -10.5052558 | 1.66E-27 | ** |
| A-B | hsa-miR-136-5p | 62 | 0 | 14.3037 | 0.01 | -10.48217268 | 4.40E-27 | ** |
| A-B | hsa-miR-337-5p | 61 | 0 | 14.073 | 0.01 | -10.45871428 | 1.17E-26 | ** |
| A-B | novel_mir_11 | 61 | 0 | 14.073 | 0.01 | -10.45871428 | 1.17E-26 | ** |
| A-B | novel_mir_12 | 61 | 0 | 14.073 | 0.01 | -10.45871428 | 1.17E-26 | ** |
| A-B | hsa-miR-20a-3p | 60 | 0 | 13.8423 | 0.01 | -10.43486796 | 3.09E-26 | ** |
| A-B | hsa-miR-3605-5p | 60 | 0 | 13.8423 | 0.01 | -10.43486796 | 3.09E-26 | ** |
| A-B | hsa-miR-376c-3p | 60 | 0 | 13.8423 | 0.01 | -10.43486796 | 3.09E-26 | ** |
| A-B | hsa-miR-19b-1-5p | 59 | 0 | 13.6116 | 0.01 | -10.41062093 | 8.18E-26 | ** |
| A-B | hsa-miR-210-5p | 59 | 0 | 13.6116 | 0.01 | -10.41062093 | 8.18E-26 | ** |
| A-B | hsa-miR-92b-3p | 58 | 0 | 13.3809 | 0.01 | -10.38595941 | 2.17E-25 | ** |
| A-B | novel_mir_13 | 58 | 0 | 13.3809 | 0.01 | -10.38595941 | 2.17E-25 | ** |
| A-B | novel_mir_14 | 56 | 0 | 12.9195 | 0.01 | -10.33533445 | 1.52E-24 | ** |
| A-B | novel_mir_15 | 53 | 0 | 12.2274 | 0.01 | -10.25590187 | 2.83E-23 | ** |
| A-B | novel_mir_16 | 53 | 0 | 12.2274 | 0.01 | -10.25590187 | 2.83E-23 | ** |
| A-B | hsa-miR-501-5p | 48 | 0 | 11.0739 | 0.01 | -10.11294773 | 3.69E-21 | ** |
| A-B | hsa-miR-1197 | 43 | 0 | 9.9203 | 0.01 | -9.95423998 | 4.82E-19 | ** |
| A-B | hsa-miR-6877-5p | 84 | 1 | 19.3793 | 0.1399 | -7.11397669 | 1.16E-34 | ** |
| A-B | hsa-miR-5706 | 79 | 1 | 18.2257 | 0.1399 | -7.02543446 | 1.43E-32 | ** |
| A-B | hsa-miR-340-3p | 54 | 1 | 12.4581 | 0.1399 | -6.47654428 | 3.76E-22 | ** |
| A-B | hsa-miR-190a-5p | 223 | 101 | 51.4473 | 14.1278 | -1.86455854 | 1.04E-29 | ** |
| A-B | hsa-miR-941 | 423 | 195 | 97.5884 | 27.2764 | -1.83905642 | 8.53E-54 | ** |
| A-B | hsa-miR-1908-5p | 156 | 77 | 35.99 | 10.7707 | -1.74048409 | 1.97E-19 | ** |
| A-B | hsa-miR-379-5p | 142 | 80 | 32.7602 | 11.1903 | -1.54969545 | 2.88E-15 | ** |
| A-B | hsa-miR-146a-5p | 129523 | 73032 | 29881.6574 | 10215.6316 | -1.54848177 | < 0.001 | ** |
| A-B | hsa-miR-29c-3p | 145 | 83 | 33.4523 | 11.6099 | -1.52674986 | 3.03E-15 | ** |
| A-B | hsa-miR-18a-5p | 669 | 387 | 154.3419 | 54.1331 | -1.51154686 | 1.86E-63 | ** |
| A-B | hsa-miR-134-5p | 341 | 211 | 78.6705 | 29.5144 | -1.41440374 | 3.71E-30 | ** |
| A-B | hsa-miR-345-5p | 139 | 88 | 32.0681 | 12.3093 | -1.38139016 | 8.39E-13 | ** |
| A-B | hsa-miR-183-5p | 140 | 98 | 32.2988 | 13.7081 | -1.23645194 | 4.74E-11 | ** |
| A-B | hsa-miR-100-5p | 374 | 280 | 86.2838 | 39.1661 | -1.13948424 | 8.37E-24 | ** |
| A-B | hsa-miR-24-3p | 1955 | 1483 | 451.0291 | 207.4403 | -1.12052432 | 7.58E-114 | ** |
| A-B | hsa-miR-151a-3p | 46918 | 36527 | 10824.2366 | 5109.3545 | -1.08305234 | < 0.001 | ** |
| A-B | hsa-miR-454-5p | 115 | 92 | 26.5311 | 12.8689 | -1.04379575 | 2.06E-07 | ** |
| A-B | hsa-miR-4446-3p | 131 | 107 | 30.2224 | 14.967 | -1.01383316 | 6.31E-08 | ** |
| A-B | hsa-miR-142-3p | 472 | 1557 | 108.893 | 217.7914 | 1.00003577 | 4.13E-44 | ** |
| A-B | hsa-miR-15a-5p | 475 | 1583 | 109.5851 | 221.4282 | 1.01478732 | 7.41E-46 | ** |
| A-B | hsa-miR-766-3p | 251 | 840 | 57.9071 | 117.4982 | 1.0208265 | 2.56E-25 | ** |
| A-B | hsa-let-7d-3p | 872 | 3008 | 201.1751 | 420.7556 | 1.06453072 | 2.77E-92 | ** |
| A-B | hsa-miR-495-3p | 132 | 459 | 30.4531 | 64.2044 | 1.07608307 | 1.16E-15 | ** |
| A-B | hsa-miR-30d-5p | 10992 | 38488 | 2535.9139 | 5383.6569 | 1.08608071 | < 0.001 | ** |
| A-B | hsa-miR-122-5p | 1403 | 4946 | 323.6797 | 691.8408 | 1.09587321 | 1.39E-157 | ** |
| A-B | hsa-miR-382-5p | 79 | 282 | 18.2257 | 39.4458 | 1.11389747 | 1.19E-10 | ** |
| A-B | hsa-miR-2355-5p | 51 | 183 | 11.766 | 25.5978 | 1.12139588 | 1.96E-07 | ** |
| A-B | hsa-miR-548k | 40 | 146 | 9.2282 | 20.4223 | 1.14602418 | 2.37E-06 | ** |
| A-B | hsa-miR-30e-5p | 3907 | 14269 | 901.3661 | 1995.9312 | 1.14687689 | < 0.001 | ** |
| A-B | hsa-miR-30a-5p | 3079 | 11247 | 710.342 | 1573.2173 | 1.14713226 | < 0.001 | ** |
| A-B | hsa-miR-1307-5p | 798 | 2934 | 184.1029 | 410.4045 | 1.1565342 | 1.80E-102 | ** |
| A-B | hsa-miR-589-5p | 447 | 1671 | 103.1253 | 233.7375 | 1.1804909 | 5.31E-61 | ** |
| A-B | hsa-miR-342-3p | 1440 | 5431 | 332.2158 | 759.682 | 1.19327495 | 2.17E-197 | ** |
| A-B | hsa-miR-374a-3p | 151 | 578 | 34.8365 | 80.85 | 1.21464809 | 4.42E-23 | ** |
| A-B | hsa-miR-4677-3p | 342 | 1310 | 78.9013 | 183.2413 | 1.21562373 | 2.32E-50 | ** |
| A-B | hsa-miR-16-5p | 2873 | 11195 | 662.8167 | 1565.9436 | 1.24035039 | < 0.001 | ** |
| A-B | hsa-miR-324-5p | 64 | 257 | 14.7651 | 35.9489 | 1.2837565 | 6.63E-12 | ** |
| A-B | hsa-miR-625-5p | 59 | 238 | 13.6116 | 33.2912 | 1.29030421 | 3.36E-11 | ** |
| A-B | hsa-miR-98-5p | 2341 | 9510 | 540.0814 | 1330.2478 | 1.30044625 | < 0.001 | ** |
| A-B | hsa-miR-425-5p | 14620 | 59862 | 3372.9132 | 8373.4273 | 1.31182306 | < 0.001 | ** |
| A-B | hsa-miR-339-3p | 192 | 795 | 44.2954 | 111.2037 | 1.327976 | 1.75E-35 | ** |
| A-B | hsa-miR-27b-5p | 76 | 316 | 17.5336 | 44.2017 | 1.33397962 | 4.32E-15 | ** |
| A-B | hsa-miR-320b | 158 | 661 | 36.4515 | 92.4599 | 1.34284962 | 3.03E-30 | ** |
| A-B | hsa-miR-374b-5p | 336 | 1421 | 77.517 | 198.7678 | 1.35849942 | 3.21E-64 | ** |
| A-B | hsa-miR-128-1-5p | 55 | 236 | 12.6888 | 33.0114 | 1.37940869 | 3.84E-12 | ** |
| A-B | hsa-miR-409-3p | 6229 | 26762 | 1437.064 | 3743.4376 | 1.38123939 | < 0.001 | ** |
| A-B | hsa-miR-190b | 47 | 203 | 10.8432 | 28.3954 | 1.38886665 | 1.00E-10 | ** |
| A-B | hsa-miR-197-3p | 271 | 1186 | 62.5212 | 165.8963 | 1.40786434 | 9.91E-57 | ** |
| A-B | hsa-miR-143-3p | 101905 | 450001 | 23510.0352 | 62945.619 | 1.42082927 | < 0.001 | ** |
| A-B | hsa-miR-451a | 10456 | 46563 | 2412.2558 | 6513.1785 | 1.43297886 | < 0.001 | ** |
| A-B | hsa-miR-181a-2-3p | 987 | 4435 | 227.7062 | 620.3627 | 1.44593837 | 1.95E-215 | ** |
| A-B | hsa-let-7a-5p | 13181 | 60030 | 3040.9281 | 8396.9269 | 1.46534972 | < 0.001 | ** |
| A-B | hsa-miR-423-3p | 841 | 3968 | 194.0233 | 555.0392 | 1.51635975 | 1.19E-206 | ** |
| A-B | hsa-miR-769-5p | 777 | 3670 | 179.2581 | 513.3554 | 1.51791965 | 1.47E-191 | ** |
| A-B | hsa-miR-652-5p | 60 | 288 | 13.8423 | 40.2851 | 1.54116266 | 8.26E-17 | ** |
| A-B | hsa-miR-377-5p | 64 | 314 | 14.7651 | 43.922 | 1.57275262 | 1.09E-18 | ** |
| A-B | hsa-miR-574-3p | 71 | 353 | 16.3801 | 49.3772 | 1.59190086 | 3.63E-21 | ** |
| A-B | hsa-miR-370-3p | 89 | 445 | 20.5328 | 62.2461 | 1.60005307 | 1.78E-26 | ** |
| A-B | hsa-miR-146b-3p | 58 | 296 | 13.3809 | 41.4041 | 1.62959848 | 1.57E-18 | ** |
| A-B | hsa-miR-501-3p | 51 | 262 | 11.766 | 36.6483 | 1.63912234 | 1.09E-16 | ** |
| A-B | hsa-miR-130b-3p | 136 | 731 | 31.3759 | 102.2514 | 1.70439185 | 2.94E-46 | ** |
| A-B | hsa-miR-411-5p | 150 | 823 | 34.6058 | 115.1203 | 1.7340565 | 4.46E-53 | ** |
| A-B | hsa-let-7b-5p | 1574 | 8642 | 363.1303 | 1208.833 | 1.73505573 | < 0.001 | ** |
| A-B | hsa-miR-654-5p | 73 | 402 | 16.8415 | 56.2313 | 1.73935276 | 7.96E-27 | ** |
| A-B | hsa-miR-432-5p | 132 | 755 | 30.4531 | 105.6085 | 1.79406495 | 4.73E-51 | ** |
| A-B | hsa-miR-433-3p | 126 | 723 | 29.0689 | 101.1324 | 1.79869689 | 4.03E-49 | ** |
| A-B | hsa-let-7d-5p | 1429 | 8661 | 329.678 | 1211.4907 | 1.87765381 | < 0.001 | ** |
| A-B | hsa-miR-210-3p | 57 | 349 | 13.1502 | 48.8177 | 1.89231958 | 4.24E-26 | ** |
| A-B | hsa-miR-889-3p | 377 | 2357 | 86.9759 | 329.6944 | 1.92244177 | 1.78E-170 | ** |
| A-B | hsa-miR-363-3p | 62 | 400 | 14.3037 | 55.9515 | 1.96778843 | 3.68E-31 | ** |
| A-B | hsa-miR-660-5p | 3 | 103 | 0.6921 | 14.4075 | 4.3796957 | 8.78E-18 | ** |
| A-B | hsa-miR-6511a-3p | 2 | 84 | 0.4614 | 11.7498 | 4.67047439 | 3.54E-15 | ** |
| A-B | hsa-miR-215-5p | 1 | 115 | 0.2307 | 16.0861 | 6.12365278 | 1.19E-22 | ** |
| A-B | hsa-miR-7706 | 1 | 266 | 0.2307 | 37.2078 | 7.33344328 | 2.25E-53 | ** |
| A-B | hsa-miR-500a-3p | 1 | 398 | 0.2307 | 55.6718 | 7.91478492 | 2.27E-80 | ** |
| A-B | hsa-miR-1260b | 0 | 62 | 0.01 | 8.6725 | 9.76030413 | 2.16E-13 | ** |
| A-B | hsa-miR-5010-5p | 0 | 69 | 0.01 | 9.6516 | 9.91462432 | 7.82E-15 | ** |
| A-B | hsa-miR-3667-3p | 0 | 74 | 0.01 | 10.351 | 10.01555444 | 7.31E-16 | ** |
| A-B | novel_mir_32 | 0 | 75 | 0.01 | 10.4909 | 10.03492273 | 4.55E-16 | ** |
| A-B | hsa-miR-320d | 0 | 77 | 0.01 | 10.7707 | 10.0728963 | 1.76E-16 | ** |
| A-B | hsa-miR-589-3p | 0 | 78 | 0.01 | 10.9105 | 10.0915015 | 1.10E-16 | ** |
| A-B | hsa-miR-3942-5p | 0 | 81 | 0.01 | 11.3302 | 10.14595761 | 2.65E-17 | ** |
| A-B | hsa-miR-6730-5p | 0 | 82 | 0.01 | 11.4701 | 10.16366225 | 1.65E-17 | ** |
| A-B | hsa-miR-412-5p | 0 | 84 | 0.01 | 11.7498 | 10.19842048 | 6.39E-18 | ** |
| A-B | hsa-miR-550b-3p | 0 | 85 | 0.01 | 11.8897 | 10.2154966 | 3.98E-18 | ** |
| A-B | hsa-miR-4707-3p | 0 | 86 | 0.01 | 12.0296 | 10.23237296 | 2.48E-18 | ** |
| A-B | hsa-miR-4742-3p | 0 | 87 | 0.01 | 12.1695 | 10.24905418 | 1.54E-18 | ** |
| A-B | hsa-miR-6515-5p | 0 | 87 | 0.01 | 12.1695 | 10.24905418 | 1.54E-18 | ** |
| A-B | novel_mir_29 | 0 | 89 | 0.01 | 12.4492 | 10.28183732 | 5.98E-19 | ** |
| A-B | novel_mir_30 | 0 | 89 | 0.01 | 12.4492 | 10.28183732 | 5.98E-19 | ** |
| A-B | novel_mir_31 | 0 | 89 | 0.01 | 12.4492 | 10.28183732 | 5.98E-19 | ** |
| A-B | hsa-miR-149-5p | 0 | 90 | 0.01 | 12.5891 | 10.29795943 | 3.72E-19 | ** |
| A-B | hsa-miR-5582-3p | 0 | 90 | 0.01 | 12.5891 | 10.29795943 | 3.72E-19 | ** |
| A-B | hsa-miR-1285-3p | 0 | 91 | 0.01 | 12.729 | 10.31390337 | 2.32E-19 | ** |
| A-B | hsa-miR-4662a-3p | 0 | 91 | 0.01 | 12.729 | 10.31390337 | 2.32E-19 | ** |
| A-B | hsa-miR-6721-5p | 0 | 92 | 0.01 | 12.8689 | 10.32967303 | 1.44E-19 | ** |
| A-B | novel_mir_28 | 0 | 92 | 0.01 | 12.8689 | 10.32967303 | 1.44E-19 | ** |
| A-B | hsa-miR-4435 | 0 | 93 | 0.01 | 13.0087 | 10.34526108 | 8.98E-20 | ** |
| A-B | hsa-miR-4687-5p | 0 | 93 | 0.01 | 13.0087 | 10.34526108 | 8.98E-20 | ** |
| A-B | hsa-miR-424-5p | 0 | 94 | 0.01 | 13.1486 | 10.36069348 | 5.59E-20 | ** |
| A-B | hsa-miR-532-3p | 0 | 94 | 0.01 | 13.1486 | 10.36069348 | 5.59E-20 | ** |
| A-B | novel_mir_27 | 0 | 94 | 0.01 | 13.1486 | 10.36069348 | 5.59E-20 | ** |
| A-B | hsa-miR-4742-5p | 0 | 95 | 0.01 | 13.2885 | 10.37596255 | 3.48E-20 | ** |
| A-B | hsa-miR-129-5p | 0 | 96 | 0.01 | 13.4284 | 10.3910717 | 2.17E-20 | ** |
| A-B | hsa-miR-3928-3p | 0 | 96 | 0.01 | 13.4284 | 10.3910717 | 2.17E-20 | ** |
| A-B | novel_mir_26 | 0 | 96 | 0.01 | 13.4284 | 10.3910717 | 2.17E-20 | ** |
| A-B | hsa-miR-25-5p | 0 | 98 | 0.01 | 13.7081 | 10.42081291 | 8.39E-21 | ** |
| A-B | hsa-miR-125b-5p | 0 | 99 | 0.01 | 13.848 | 10.43546191 | 5.23E-21 | ** |
| A-B | hsa-miR-1296-5p | 0 | 99 | 0.01 | 13.848 | 10.43546191 | 5.23E-21 | ** |
| A-B | hsa-miR-491-5p | 0 | 99 | 0.01 | 13.848 | 10.43546191 | 5.23E-21 | ** |
| A-B | hsa-miR-671-3p | 0 | 99 | 0.01 | 13.848 | 10.43546191 | 5.23E-21 | ** |
| A-B | hsa-miR-6770-3p | 0 | 99 | 0.01 | 13.848 | 10.43546191 | 5.23E-21 | ** |
| A-B | novel_mir_25 | 0 | 99 | 0.01 | 13.848 | 10.43546191 | 5.23E-21 | ** |
| A-B | hsa-miR-1278 | 0 | 100 | 0.01 | 13.9879 | 10.44996367 | 3.25E-21 | ** |
| A-B | hsa-miR-200a-5p | 0 | 100 | 0.01 | 13.9879 | 10.44996367 | 3.25E-21 | ** |
| A-B | hsa-miR-431-3p | 0 | 100 | 0.01 | 13.9879 | 10.44996367 | 3.25E-21 | ** |
| A-B | hsa-miR-1287-5p | 0 | 101 | 0.01 | 14.1278 | 10.46432111 | 2.03E-21 | ** |
| A-B | hsa-miR-376a-5p | 0 | 101 | 0.01 | 14.1278 | 10.46432111 | 2.03E-21 | ** |
| A-B | novel_mir_24 | 0 | 101 | 0.01 | 14.1278 | 10.46432111 | 2.03E-21 | ** |
| A-B | hsa-miR-485-3p | 0 | 102 | 0.01 | 14.2676 | 10.47852696 | 1.26E-21 | ** |
| A-B | hsa-miR-548e-5p | 0 | 102 | 0.01 | 14.2676 | 10.47852696 | 1.26E-21 | ** |
| A-B | hsa-miR-365a-3p | 0 | 103 | 0.01 | 14.4075 | 10.4926043 | 7.85E-22 | ** |
| A-B | hsa-miR-365b-3p | 0 | 103 | 0.01 | 14.4075 | 10.4926043 | 7.85E-22 | ** |
| A-B | hsa-let-7g-3p | 0 | 104 | 0.01 | 14.5474 | 10.50654561 | 4.89E-22 | ** |
| A-B | hsa-miR-3074-5p | 0 | 104 | 0.01 | 14.5474 | 10.50654561 | 4.89E-22 | ** |
| A-B | hsa-miR-338-3p | 0 | 104 | 0.01 | 14.5474 | 10.50654561 | 4.89E-22 | ** |
| A-B | hsa-miR-4762-5p | 0 | 104 | 0.01 | 14.5474 | 10.50654561 | 4.89E-22 | ** |
| A-B | hsa-miR-758-3p | 0 | 104 | 0.01 | 14.5474 | 10.50654561 | 4.89E-22 | ** |
| A-B | novel_mir_23 | 0 | 105 | 0.01 | 14.6873 | 10.52035349 | 3.04E-22 | ** |
| A-B | hsa-miR-1538 | 0 | 106 | 0.01 | 14.8272 | 10.53403047 | 1.89E-22 | ** |
| A-B | hsa-miR-191-3p | 0 | 106 | 0.01 | 14.8272 | 10.53403047 | 1.89E-22 | ** |
| A-B | novel_mir_22 | 0 | 106 | 0.01 | 14.8272 | 10.53403047 | 1.89E-22 | ** |
| A-B | hsa-miR-3613-3p | 0 | 108 | 0.01 | 15.1069 | 10.56099193 | 7.34E-23 | ** |
| A-B | hsa-miR-543 | 0 | 109 | 0.01 | 15.2468 | 10.57429077 | 4.57E-23 | ** |
| A-B | hsa-miR-3074-3p | 0 | 110 | 0.01 | 15.3867 | 10.58746813 | 2.84E-23 | ** |
| A-B | hsa-miR-487a-3p | 0 | 110 | 0.01 | 15.3867 | 10.58746813 | 2.84E-23 | ** |
| A-B | hsa-miR-15b-3p | 0 | 111 | 0.01 | 15.5266 | 10.60052623 | 1.77E-23 | ** |
| A-B | novel_mir_21 | 0 | 111 | 0.01 | 15.5266 | 10.60052623 | 1.77E-23 | ** |
| A-B | hsa-miR-4775 | 0 | 112 | 0.01 | 15.6664 | 10.61345798 | 1.10E-23 | ** |
| A-B | novel_mir_20 | 0 | 117 | 0.01 | 16.3658 | 10.67646841 | 1.03E-24 | ** |
| A-B | hsa-miR-656-3p | 0 | 118 | 0.01 | 16.5057 | 10.68874861 | 6.42E-25 | ** |
| A-B | novel_mir_19 | 0 | 148 | 0.01 | 20.7021 | 11.01556141 | 4.29E-31 | ** |
| A-B | hsa-miR-33b-5p | 0 | 164 | 0.01 | 22.9401 | 11.16365596 | 2.18E-34 | ** |
| A-B | novel_mir_18 | 0 | 180 | 0.01 | 25.1782 | 11.29795943 | 1.11E-37 | ** |
| A-B | hsa-miR-4645-3p | 0 | 184 | 0.01 | 25.7377 | 11.32966742 | 1.67E-38 | ** |
| A-B | hsa-miR-4799-5p | 0 | 187 | 0.01 | 26.1573 | 11.35299792 | 4.03E-39 | ** |
| A-B | hsa-miR-30c-1-3p | 0 | 192 | 0.01 | 26.8567 | 11.39106633 | 3.77E-40 | ** |
| A-B | hsa-miR-1468-5p | 0 | 194 | 0.01 | 27.1365 | 11.40601894 | 1.46E-40 | ** |
| A-B | hsa-miR-17-3p | 0 | 196 | 0.01 | 27.4163 | 11.42081817 | 5.66E-41 | ** |
| A-B | hsa-miR-93-3p | 0 | 198 | 0.01 | 27.696 | 11.43546191 | 2.19E-41 | ** |
| A-B | hsa-miR-30c-2-3p | 0 | 202 | 0.01 | 28.2555 | 11.464316 | 3.29E-42 | ** |
| A-B | hsa-miR-421 | 0 | 211 | 0.01 | 29.5144 | 11.5272033 | 4.63E-44 | ** |
| A-B | hsa-miR-378c | 0 | 214 | 0.01 | 29.9341 | 11.54757418 | 1.12E-44 | ** |
| A-B | hsa-miR-99b-3p | 0 | 221 | 0.01 | 30.9132 | 11.59400729 | 4.05E-46 | ** |
| A-B | hsa-miR-320c | 0 | 223 | 0.01 | 31.193 | 11.6070066 | 1.57E-46 | ** |
| A-B | hsa-miR-3613-5p | 0 | 228 | 0.01 | 31.8924 | 11.63899695 | 1.47E-47 | ** |
| A-B | hsa-miR-331-3p | 0 | 238 | 0.01 | 33.2912 | 11.70092516 | 1.28E-49 | ** |
| A-B | hsa-miR-577 | 0 | 269 | 0.01 | 37.6274 | 11.87756789 | 5.34E-56 | ** |
| A-B | hsa-miR-145-3p | 0 | 283 | 0.01 | 39.5857 | 11.95076365 | 7.01E-59 | ** |
| A-B | hsa-miR-22-5p | 0 | 298 | 0.01 | 41.6839 | 12.02527455 | 5.73E-62 | ** |
| A-B | hsa-miR-144-3p | 0 | 375 | 0.01 | 52.4546 | 12.35685358 | 8.11E-78 | ** |
| A-B | hsa-miR-29a-3p | 0 | 476 | 0.01 | 66.5823 | 12.70092299 | 1.32E-98 | ** |
| A-B | hsa-miR-144-5p | 0 | 574 | 0.01 | 80.2905 | 12.97101358 | 8.90E-119 | ** |
| A-B | novel_mir_17 | 0 | 7743 | 0.01 | 1083.0819 | 16.72478281 | < 0.001 | ** |
| *Abbreviation: A represents LOH group; B represents health control group. | | | | | | | | |

**Table S2. Cq values of miRNAs in fq-RT-PCR in the training phase*.**

| **sample** | **miR-125a-5p** | **miR-361-5p** | **miR-150-5p** | **miR-133a-3p** | **miR-1301-3p** | **let-7b-5p** | **let-7i-3p** | **U6** |
| --- | --- | --- | --- | --- | --- | --- | --- | --- |
| A1 | 33.84 | 34.54 | 36.09 | 35.72 | 29.71 | 25.82 | 40.42 | 34.49 |
| A2 | 31.34 | 31.66 | 33.43 | 34.66 | 28.29 | 22.97 | 36.54 | 31.17 |
| A3 | 30.6 | 32.58 | 29.59 | 34.27 | 27.01 | 21.82 | 35.89 | 32.01 |
| A4 | 32.65 | 33.97 | 33.9 | 36.29 | 28.98 | 26.45 | 39.47 | 33.86 |
| A5 | 32.26 | 31.35 | 29.79 | 33.84 | 27.38 | 23.67 | 36.13 | 31.66 |
| A6 | 30.43 | 31.14 | 31.08 | 32.9 | 26.42 | 22.52 | 34.67 | 33.24 |
| A7 | 32.34 | 34.3 | 35.41 | 36.75 | 31.59 | 24.43 | 38.73 | 34.25 |
| A8 | 31.58 | 31.95 | 29.36 | 34.75 | 28.51 | 23.32 | 35.58 | 32.06 |
| A9 | 33.85 | 33.49 | 30.55 | 32.74 | 27.19 | 22.09 | 34.32 | 31.45 |
| A10 | 29.93 | 32.36 | 32.87 | 34.97 | 27.29 | 23.32 | 35.96 | 31.66 |
| B1 | 27.99 | 30.38 | 27.64 | 33.22 | 28.19 | 21.94 | 36.39 | 31.45 |
| B2 | 29.43 | 32.81 | 31.53 | 34.34 | 29.49 | 24.72 | 36.98 | 32.63 |
| B3 | 28.48 | 29.71 | 31.48 | 31.47 | 31.26 | 22.4 | 37.12 | 31.58 |
| B4 | 27.97 | 29.73 | 29.63 | 32.8 | 29.63 | 22.78 | 35.02 | 31.72 |
| B5 | 32.25 | 30.36 | 28.65 | 31.23 | 27.79 | 23.93 | 35.36 | 33.24 |
| B6 | 28.1 | 31.08 | 31.4 | 32.47 | 30.61 | 22.01 | 36.08 | 32.23 |
| B7 | 28.96 | 30.68 | 28.21 | 32.9 | 28.85 | 22.57 | 34.73 | 32.19 |
| B8 | 31.94 | 32.51 | 29.26 | 33.54 | 29.46 | 25.12 | 35.21 | 32.99 |
| B9 | 30.17 | 29.79 | 31.18 | 31.63 | 28.44 | 23.22 | 35.05 | 31.48 |
| B10 | 27.38 | 30.51 | 28.64 | 31.22 | 28.59 | 22.06 | 36.52 | 31.23 |
| *Abbreviation: A represents LOH group; B represents health control group. U6, samll nuclear RNA U6. | | | | | | | | |

**Table S3. The primer sequences of selected miRNAs in discovery phase for real-time PCR.**

| miRNA | Sequences |
| --- | --- |
| hsa-miR-3615 | TCTCTCGGCTCCTCGCGGCTC |
| hsa-miR-4433b-3p | TCAGTGCATCACAGAACTTTGT |
| hsa-miR-99a-5p | AACCCGTAGATCCGATCTTGTG |
| hsa-miR-148b-3p | TCAGTGCATCACAGAACTTTGT |
| hsa-miR-1301-3p | TTGCAGCTGCCTGGGAGTGACTTC |
| hsa-miR-150-5p | TCTCCCAACCCTTGTACCAGTG |
| hsa-miR-106b-5p | TAAAGTGCTGACAGTGCAGAT |
| hsa-miR-125a-5p | TCCCTGAGACCCTTTAACCTGTGA |
| hsa-miR-335-5p | TCAAGAGCAATAACGAAAAATGT |
| hsa-miR-877-5p | GTAGAGGAGATGGCGCAGGG |
| hsa-miR-505-3p | CGTCAACACTTGCTGGTTTCCT |
| hsa-miR-7849-3p | GACAATTGTTGATCTTGGGCCT |
| hsa-miR-133a-3p | TTTGGTCCCCTTCAACCAGCTG |
| hsa-miR-361-5p | TTATCAGAATCTCCAGGGGTAC |
| hsa-let-7e-5p | TGAGGTAGGAGGTTGTATAGTT |
| hsa-miR-381-3p | TATACAAGGGCAAGCTCTCTGT |
